# Evolution of rehabilitation services in response to a global pandemic: reflection on opportunities and challenges ahead

**DOI:** 10.3389/fresc.2023.1173558

**Published:** 2023-05-15

**Authors:** Fransiska M. Bossuyt, Yelena Bogdanova, Kristine T. Kingsley, Thomas F. Bergquist, Stephanie A. Kolakowsky-Hayner, Zaliha Binti Omar, Evguenia S. Popova, Mari Tobita, Fofi Constantinidou

**Affiliations:** ^1^ Neuro-musculoskeletal Functioning and Mobility Group, Swiss Paraplegic Research, Nottwil, Switzerland; ^2^ Department of Psychiatry, Boston University School of Medicine, Boston, MA, United States; ^3^ Physical Medicine & Rehabilitation, VA Boston Healthcare System, Boston, MA, United States; ^4^ Institute of Emotional and Cognitive Wellness, New York, NY, United States; ^5^ Department of Psychology, Teachers College, Columbia University, New York, NY, United States; ^6^ Department of Physical Medicine and Rehabilitation, Mayo Clinic, Rochester, MN, United States; ^7^ Department of Research and Clinical Outcomes, Good Shepherd Rehabilitation Network, Allentown, PA, United States; ^8^ Department of Rehabilitation Medicine, University Malaya, Kuala Lumpur, Malaysia; ^9^ Department of Rehabilitation Medicine 1, Fujita Health University, Aichi, Japan; ^10^ Department of Occupational Therapy, Rush University, Chicago, IL, United States; ^11^ Rancho Los Amigos National Rehabilitation Center, Downey, CA, United States; ^12^ Rancho Research Institute, Downey, CA, United States; ^13^ Center for Applied Neuroscience & Department of Psychology, University of Cyprus, Nicosia, Cyprus

**Keywords:** pandemic, rehabilitation, COVID-19, long term care facilities, telerehabilitation

## Abstract

The rapidly evolving COVID-19 public health emergency has disrupted and challenged traditional healthcare, rehabilitation services, and treatment delivery worldwide. This perspective paper aimed to unite experiences and perspectives from an international group of rehabilitation providers while reflecting on the lessons learned from the challenges and opportunities raised during the COVID-19 pandemic. We discuss the global appreciation for rehabilitation services and changes in access to healthcare, including virtual, home-based rehabilitation, and long-term care rehabilitation. We illustrate lessons learned by highlighting successful rehabilitation approaches from the US, Belgium, and Japan.

## Introduction

One in every three people will need rehabilitation services at some point in their lifetime (1). Yet, not only rehabilitation services remain underappreciated and under-resourced, the ageing population and increase in non-communicable conditions resulted in a significant increase in absolute physical rehabilitation needs of 66% worldwide between 1990 and 2017 (2). Importantly, this increase was nearly twice as high with 112% for low-income countries which are expected to have underdeveloped rehabilitation services (2). More specifically, Asia-Pacific, Latin America & Caribbean, as well as South Asia and Sub-Saharan Africa regions presented greatest changes in the absolute, relative, and percentage of physical rehabilitation needs (3). The Rehabilitation 2030 Initiative by the World Health Organization (WHO) draws attention to the profound and global unmet need for rehabilitation services (4). The COVID-19 pandemic further disrupted and challenged traditional healthcare, rehabilitation services, and treatment delivery. Furthermore, the pandemic established a new set of clinical priorities, with survivors of COVID-19 often presenting with significant rehabilitation needs, which are now being investigated by the rehabilitation community (5).

In addition to barriers to healthcare and rehabilitation services (6–8), the implementation of social distancing measures through various phases of the pandemic imposed multiple barriers and challenges for already vulnerable populations, including the elderly, women, economically disadvantaged, racial and ethnic minorities, uninsured, homeless, and the disability communities (9). Individual vulnerability combined with the lack of timely access to healthcare may have led to and exacerbated the disproportionate health risks experienced by people with disabilities during the COVID-19 pandemic (10). Furthermore, women were more likely to experience psychological disorders and be subjected to intimate partner violence because of quarantine (11, 12). The COVID-19 pandemic has robustly affected global mental health and highlighted the importance of mental health care services. Specifically, a meta-analysis of 20 studies on psychological issues suggested an overall prevalence of symptoms such as anxiety and depression among the general population ranging from 28% to 36% (13). The negative physical and mental health outcomes associated with COVID-19, stressed the importance of timely quality care for patients within vulnerable communities, including people with disabilities and other pre-existing health conditions who were at a higher risk of infection (14, 15). Despite the barriers that emerged during the pandemic, changes to care and rehabilitation were also found to be a facilitator affecting the lives of vulnerable populations through for example new innovations (7).

Although the COVID-19 pandemic resulted in an accelerated publication rate in rehabilitation, with 18% of all publications published between 2019 and 2022 including the term “rehabilitation” (Pubmed, Jan 2023: 133.534), only recently, there has been a first collection of publications addressing the challenges and opportunities of health systems, rehabilitation care, and COVID-19 (16). Therefore, this perspective paper aims to unite findings, experiences, and perspectives from an international group of rehabilitation providers on the challenges and opportunities resulting from the COVID-19 pandemic.

## Challenges and opportunities ahead

### A new diagnosis

In January 2023, COVID-19 was diagnosed in more than 660 million individuals in 229 countries and territories around the globe, resulting in 6.7 million deaths and over 638 million recoveries (17). Though men and women are reported to contract COVID-19 at similar rates, gender differences have been noted in the prognosis. While men are reported to have higher morbidity and mortality (18–20) and a more extensive lung disease process (21); women are more likely to be affected by the lingering effects of COVID-19 and with long-COVID syndrome, otherwise known as long-COVID (22–25). Long-COVID refers to a constellation of symptoms present three months after the onset of COVID-19 symptomatology and persisting for at least two months (26), and presents in a new type of disability for healthcare and rehabilitation providers. A meta-analysis demonstrated that over 20% of COVID-19 patients displayed fatigue or cognitive impairment at 12 weeks post-infection, regardless of infection severity or hospitalization (27). Researchers across the globe continue their work to characterize the outcomes (23, 24, 28) and causes of these symptoms, but the severe immune response to COVID-19 seems to be one of the leading causes. Large consortia have been created, and many government agencies supported initiatives to prospectively study the course of long-COVID. While research efforts are underway across the globe, the U.S. Veterans Health Administration (VHA) has established more than 20 long-COVID programs, providing multidisciplinary care for veterans with long-COVID, as well as a Long-COVID Community of Practice connecting clinicians leading efforts to care for veterans with long-COVID (29). Of interest, the community has been investigating the emerging neurobehavioral phenotypes, including post-traumatic stress disorder, physical and mental fatigue, and neurocognitive dysfunction. Growing knowledge about the long-term impact of COVID-19 calls for ongoing research and knowledge translation of novel rehabilitation approaches designed to support COVID-19 recovery (30).

## Responses to COVID-19 across the globe

Differences in how countries responded to the pandemic and adjusted their rehabilitation services demonstrate variability in healthcare systems and priorities (31, 32). For example, following recommendations from the Centers for Disease Control and Prevention (CDC) and the WHO, governments in Europe, North/South America, Africa, and Asia including 12 low-income, middle-income and high-income countries tried to reduce the duration of inpatient treatments (31). To this effect, a scoping review with studies from different countries including most commonly the US, the U.K., and Brazil, showed significant disruption to healthcare during the pandemic and worsening health outcomes in persons with disabilities (7). In Germany, the pandemic caused a reduction in the number of medical rehabilitation requests by 14.5% (33). In a low-income country such as Jordan, where rehabilitation services in public hospitals are limited to outpatient clinics, retrospective data analysis of records of 32,503 patients between January 2020 and February 2021 showed a significant decline in those reaching rehabilitation services, reaching almost zero in May 2020, this was followed by an increase exceeding the number of patients accessing rehabilitation services prior to the onset of the pandemic (34). As a response of the second wave, the number of patients who visited the rehabilitation clinics reduced again reaching a plateau in February 2021. In South Africa, with a national healthcare system characterized by stark discrepancies between the public and private sector on account of institutional segregation policies, the vast majority of rehabilitation services were allocated to private hospitals catering to the more affluent and White populations (35). For persons with disabilities, results from 35 countries within Europe, including 99% of the population (809.9 million), showed a halt of admissions to rehabilitation, early discharge, reduction of activities in 194.800 inpatients in 10 countries, and termination of outpatient activities for 87% involving 318.000 patients per day in Italy, Belgium and the U.K. (36). In addition, over 76% of the cardiac rehabilitation programs across 70 countries in Africa, America, Eastern Mediterranean, Europe, South-East Asian and Western Pacific were stopped or ceased due to the pandemic (37).

This was not a global response; in other parts of the world rehabilitation services were deemed more of a priority. A registry-based study from Norway, including 1310 hospitalized patients with traumatic brain injury (TBI), demonstrated that the direct pathway to early specialized rehabilitation was maintained during 2020–2021 (38). Similarly, while Japan commonly follows the recommendations of the CDC and WHO, the Japanese government and the leading Medical Rehabilitation Organization did not recommend early discharge from the hospital (39, 40). Although in isolation and depending on the individual medical facility, some patients received inpatient rehabilitation until they were able to regain full independence in the community. Given Japan's universal health insurance system, an extended stay in the hospital did not result in higher costs from the point of infection control for the entire country, where the living environment is densely populated, compared to other countries. In addition, under the pre-existing Universal Health Coverage (41) and long-term care insurance (42), patients undergoing treatment for COVID-19 in Japan automatically qualified to receive rehabilitation services.

## Access to care

One of the most significant transformations in the delivery of healthcare services due to the pandemic has been innovation in remote delivery of care, including the use of telehealth. What seemed improbable pre-pandemic is now becoming an option of care currently reimbursable by insurances for individuals with limited access to physical healthcare facilities in many countries (37). Rehabilitation interventions administered in-person pre-COVID for individuals with cognitive disabilities and their caregivers are now offered remotely and with good results. Telehealth proved instrumental in rehabilitation, and offers opportunities to continue supporting healthcare access and optimize access for vulnerable populations through optimization of financial, educational, and cyber-security infrastructure (43).

Many professional associations and some government agencies across the globe [e.g., the European Speech and Language pathology association (ESLA), Government agencies and professional organizations guidance for Tele-rehab (44, 45)] are creating and publishing guidelines for remote consultation and treatment, providing online and live webcast sessions with experts to train rehabilitation providers and caregivers. In some countries, a hybrid model of service delivery (combination in-person and remote healthcare services) is becoming a standard of care. In response to the COVID-19 pandemic, members of the Task Force for research at the Indian Federation of Neurorehabilitation reviewed the context of tele-neurorehabilitation providing implications for practice of tele-neurorehabilitation in low- and middle-income countries (46). As these services continue to evolve, longitudinal health and functional outcome assessments will be essential to monitor effectiveness and support the future direction of healthcare and rehabilitation systems.

The growth of telehealth and other remote services is not only seen with COVID-19 patients but also within the healthcare system for medically vulnerable individuals and persons with limited access to healthcare in isolated areas of the world, including rural areas and parts of the world impacted by disaster and war. Telehealth has been shown to support patient-provider communication when face-to-face interaction is not possible (6). Telehealth benefits, such as improved treatment accessibility, continuous care, and opportunity for interdisciplinary rehabilitation, as well as reduced cost and travel burden, encourage the future development of telehealth-based treatment programs and home-based rehabilitation protocols.

### Telehealth and home-based rehabilitation

Telehealth, home-based rehabilitation programs, and various web-based interventions were introduced early in some medical centers in the US (47, 48) and Japan (49–51). The Neurorehab TBI Clinic at the Boston VA Healthcare System and Boston University School of Medicine was among the first to utilize the new technology for home-treatment delivery. The Virtual Care LED TBI Program provides portable neuromodulation home treatment with telehealth support for patients with chronic TBI, Post Traumatic Stress Disorder, and sleep disturbance (47, 52, 53). The Neurorehab TBI clinic was converted to virtual care immediately following the COVID-19 social distancing guidelines and continues to provide virtual clinical care to date. The patients who completed the rehabilitation program reported improved cognitive and neurobehavioral symptoms (29, 48, 54) and opted to continue the long-term home treatment program and virtual care visits even after the pandemic restrictions were lifted. Following the initial success of the home-based treatment program, the Neurorehabilitation LED TBI Clinical team expanded its services to provide virtual care to patients post TBI in 15 other states across the U.S. Furthermore, the team supported ongoing professional development by offering virtual training for the VA PMR providers across the U.S.

In Japan, dedicated virtual consultation services were introduced early in the pandemic through the Japanese Infectious Disease Prevention Act, where public health centers became responsible for infectious disease control and prevention (55, 56). An improved version of teleconsultation service, supported by the local government, was reported in Hiroshima city, and included a hotline for COVID-19 center available 24-hours a day, providing online consultation in 10 languages. The interdisciplinary team included a manager, medical doctors, nurses, and pharmacists, that could be consulted on a variety of medical needs resulting from COVID-19, ranging from interpretation of symptoms, prescription, delivery of medications, and arrangements for rehabilitation (57).

Despite the many benefits to telehealth, barriers to telehealth access were also noted. In some cases, patients who were receiving care at home did not have the resources (computers, reliable internet, and privacy) to engage in telehealth sessions. The lack of resources in low-income countries could explain why approaches of telehealth were limited in e.g., Tanzania (31). Indeed, a Cochrane qualitative review on factors that influence the provision of home-based rehabilitation services including 223 studies of which 8 were performed in low- and middle-income countries, found that despite multiple factors that facilitate home-based rehabilitation, in low-income settings in specific, worse or no internet connectivity, high technology costs, lack of technology, risk of being robbed in public spaces when using tablets, and capacity to invest in infrastructure and maintenance were barriers for home-based rehabilitation (43). These results demonstrate the importance of low- or no-cost technologies, easy-to-use technologies, as well as training and support when implementing home-based rehabilitation (43). In long-term facilities, telehealth proved hard to structure because it still required someone within the facility to set up and supervise the process. In Europe, several countries have not yet established laws to regulate telehealth, and in some countries, telehealth practice is prohibited. In an effort to address regulation barriers to telehealth access, ESLA issued a statement on the importance of telehealth in service provision (58). The Directorate General of Health, Food, and Drug in the European Union endorsed this statement. This was a significant achievement that led the way to changes in laws and regulations in Europe and had a spreading effect on other healthcare professions.

Furthermore, telehealth, was noted to not be conducive to all types of conditions and rehabilitation services. For example, in speech-language pathology, online swallowing tests were recommended only as screenings; full evaluations and interventions were discouraged. In physical therapy, requests to allow therapists to treat patients remotely were deemed impractical or even unsafe. As a result, in the U.S., Centers for Medicare & Medicaid Services motioned to deny payment for certain types of telehealth services.

During this process, many allied health professionals became strong advocates, not only for their patients but also for their profession. On several occasions, professionals took action by writing letters to Ministries of Health and introducing protocols that would inform safe practice. These actions allowed allied health professionals in Europe, for example, working with the National Health System, to notify state officials and administrators as to what rehabilitation specialty consists of and what allied health care professionals do.

## Impact on long-term care rehabilitation

Long-term care facilities (LTCFs) inhabiting vulnerable populations, including the elderly and persons with disability, are at significant risk for massive outbreaks of viruses, including COVID-19 (59). COVID-19 deaths in LTCFs including nursing homes, assisted living facilities and group homes made up over 20 percent of all COVID-19 deaths in the US (60, 61). This share has dropped over time for a variety of reasons including high rates of vaccinations among residents and staff, an increased emphasis on infection control procedures, declining nursing home occupancy, but also lack of data in LTCFs in recent months (60). While these challenges increased burden on the staff (62, 63), they also offered opportunities as presented in the success stories below.

### Success story from Belgium

Dominiek Savio is one of the most prominent institutes for more than 500 children and adults with physical disabilities in Belgium, a country in which on May 3rd 2020, 53% of all deaths due to COVID-19 were in care homes (64). Given over 80% of the population served suffer from chronic airway diseases, Dominiek Savio reacted quickly to minimize any risk of an outbreak within the institute. Their success was demonstrated over the first 4.5 months of the pandemic; with 0% of the patients served within the institute testing positive for COVID-19. Lessons learned and opportunities for rehabilitation were examined using semi-structured interviews with the COVID-19 follow-up representative and coordinating director.

At the onset of the pandemic, the board of directors selected a group of three persons that were given authority to make decisions and implement measures against the spread of the virus. The two medical doctors of the institute provided the team with the latest updates via their network. Challenges could be tackled within the organization with the support of their medical team, including 2 medical doctors, 28 nurses, and 5 healthcare providers. Because of the fast-shrinking supply of personal protective equipment (PPE), residents safely produced face masks in the workshop. Proactive actions that supported a timely response to the pandemic was the initiative taken one year before the onset of the COVID-19 pandemic, to sensitize employees on the importance of hygiene (e.g., through the availability of automatic hand disinfectants and provided instructions on the use of PPE).

Despite the lockdown, all patients received the treatments and rehabilitation they needed while considering the well-being of both patients and employees. Initiatives such as the Chatbus, i.e., a bus separated in two parts by a plastic wall allowed contact between residents and visitors. Infographics were created and distributed to allow residents to make informed decisions about vaccination and PPE. Through the pandemic, the team adjusted their strategies, and in January 2022, they reduced the burden on the staff and residents by limiting the amount of PPE to Filtering Face Piece 2 masks. The call center “Coronafoon” allowed to collect and monitor the number of positive cases and provide timely information to the leadership team. The implementation of an emergency plan with a barometer which incorporates four main principles (1. Solidarity, 2. Contextuality, 3. Differentiation, 4. Well-being of the patients and employees) gives guidance and trust for the future. The years of investment in solidarity and commitment amongst employees to improve the quality of life of persons with a disability proved its impact.

### Success story from Japan

Similar strategies were observed in Japan, which presented low mortality and morbidity rates in care homes (65). For example, the long-term care insurance introduced in 2000 had been revised and matured enough at the time of the pandemic (66). The wide range of coverage continued to care for the needs of the elderly and people with disabilities. Furthermore, standard operating procedures for rapidly spreading infections, like influenza, were already in place in nursing homes, long-term daycare facilities, and home rehabilitation services. Hence infection control measures for COVID-19 were akin to an extension of this service. Nursing homes readily implemented national policies during the pandemic through communication between residents and family members on a virtual platform. In addition, recreational activities like gardening, exercise, music, and other therapies were modified and not completely halted. Finally, access to alternative rehabilitation services was readily available in situations where a daycare center had to be closed due to a COVID cluster; users could access alternative services, including telehealth and home services.

## Discussion

The COVID-19 pandemic offered insights into how different countries across the globe prioritize rehabilitation. Those countries that were not able to provide continued rehabilitation services during the pandemic are expected to suffer from detrimental consequences, including increased rates of chronic diseases, growing healthcare costs, and reduced overall quality of life. To accommodate the patient's needs and address the challenges in the COVID-19 pandemic, rehabilitation specialists have devised innovative ways to deliver rehabilitation services for patients and caregivers. Continued research of innovative interventions and remote treatment delivery methods (including development and evaluation of the most optimal and lasting rehabilitation outcomes, capacity building of patients, caregivers, families, and providers as well as eliminating barriers to infrastructure and financing) and government support is needed to inform clinical recommendations and rehabilitation guidelines around the globe. Common findings from the presented success stories from LTCFs demonstrate the importance and effectiveness of a comprehensive approach where health care and rehabilitation are a critical part of one another as well as preparedness and having systems that can reduce the impacts of large-scale unexpected disruption to services, such as the COVID-19 pandemic.

The presented stories from high-income developed countries also align with challenges and recommendations from the low-income countries Jordan (34) and India (46), and the low-income under developed country Bangladesh (67). Although scarce, the emerging literature on low-income and under developed countries highlighted the need for multidisciplinary rehabilitation teams with scale-up of rehabilitation services (67). A recent article from South Africa reported how the consequences of discontinued, restricted or disrupted rehabilitation led to a reappraisal of the field as an essential service and highlighted the competencies of rehabilitation specialists as paramount in managing recovery and mental health needs (35).

The unpreparedness to react effectively and promptly to the pandemic was presented as one of the significant public health challenges (68). Identifying lessons learned and raising opportunities is a crucial step to improving global preparedness and ability to understand the multidimensional effects of the pandemic across social, technological, economic, and health contexts. Future research needs to identify the long-term impact of the pandemic on rehabilitation, health, and mortality across the globe and in different populations, including vulnerable populations. Rehabilitation medicine has evolved in response to the health impact of pandemics, wars, and natural disasters (69–72). On each occasion, the people around the globe were able to come together to overcome the challenges presented, and move toward advancement of rehabilitation medicine. The COVID-19 pandemic has provided the opportunity to continue evolving our approaches, and the rehabilitation community is called to continue innovating in the future.

## Data Availability

The original contributions presented in the study are included in the article, further inquiries can be directed to the corresponding author.
